# Kinetic Modeling of the Mitochondrial Energy Metabolism of Neuronal Cells: The Impact of Reduced *α*-Ketoglutarate Dehydrogenase Activities on ATP Production and Generation of Reactive Oxygen Species

**DOI:** 10.1155/2012/757594

**Published:** 2012-06-10

**Authors:** Nikolaus Berndt, Sascha Bulik, Hermann-Georg Holzhütter

**Affiliations:** Institute of Biochemistry, University Medicine-Charité, 13347 Berlin, Germany

## Abstract

Reduced activity of brain *α*-ketoglutarate dehydrogenase complex (KGDHC) occurs in a number of neurodegenerative diseases like Parkinson's disease and Alzheimer's disease. In order to quantify the relation between diminished KGDHC activity and the mitochondrial ATP generation, redox state, transmembrane potential, and generation of reactive oxygen species (ROS) by the respiratory chain (RC), we developed a detailed kinetic model. Model simulations revealed a threshold-like decline of the ATP production rate at about 60% inhibition of KGDHC accompanied by a significant increase of the mitochondrial membrane potential. By contrast, progressive inhibition of the enzyme aconitase had only little impact on these mitochondrial parameters. As KGDHC is susceptible to ROS-dependent inactivation, we also investigated the reduction state of those sites of the RC proposed to be involved in ROS production. The reduction state of all sites except one decreased with increasing degree of KGDHC inhibition suggesting an ROS-reducing effect of KGDHC inhibition. Our model underpins the important role of reduced KGDHC activity in the energetic breakdown of neuronal cells during development of neurodegenerative diseases.

## 1. Introduction

A decline in the activity of the thiamine-dependent enzyme complex *α*-ketoglutarate dehydrogenase (KGDHC) in brain has been reported for numerous age-related neurodegenerative diseases [1, 2]. In Alzheimer's disease, reductions in brain KGDHC activity range from 25 to 75% [3–7] and are strongly correlated to the decline in cognition [8]. Variations in the amounts of KGDHC between different brain regions [9, 10] may account for the brain region-specific different vulnerabilities. Neurons containing high amount of KGDHC like cholinergic neurons in the nucleus basalis are particularly susceptible to Alzheimer's disease [3, 11].

The citric acid cycle is catalyzed by eight enzymes, among which KGDHC has the lowest activity [12]. Thus, KGDHC is considered one of the rate-limiting enzymes in the tricarbonic acid cycle (TCAC). It has been proposed that reduced activity of this enzyme complex initiates a cascade of adverse processes, including metabolic failure, mitochondrial membrane depolarization, calcium overload, and cytochrome c release, eventfully leading to cell death [13]. The same cascade has been implicated in the massive death of dopaminergic neurons in the substantia nigra of patients with Parkinson's disease [14].

The molecular mechanisms underlying the age-dependent loss of brain KGDHC activity remain elusive. Inactivation of the enzyme complex by reactive oxygen species (ROS) is one possible explanation as KGDHC and the aconitase have been shown to be the main targets of ROS in the citric acid cycle [15].

To further clarify the implications of reduced activities of the TCAC enzymes KGDHC and aconitase for the mitochondrial energy metabolism and the formation of ROS by the respiratory chain, we developed and applied a detailed kinetic model encompassing the TCAC, the respiratory chain (RC), translocation of adenine nucleotides between mitochondrial matrix and the cytosol, oxidative phosphorylation, and ion transport across the inner mitochondrial membrane. The submodel of the RC describes the electron transport as a multistep process whereby some of the intermediate redox sites allow electron transfer to molecular oxygen under formation of the superoxide anion (ROS).

## 2. Model

**Figure 1:**
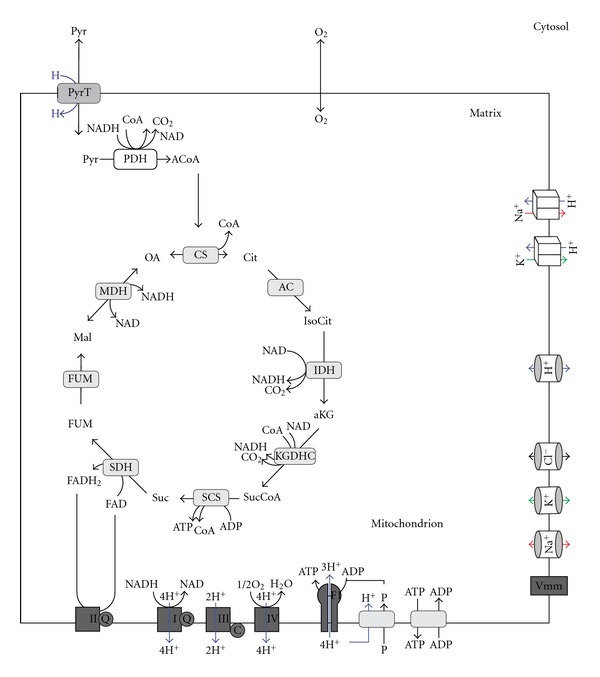
Schematic of the mathematical model. Pyruvate (Pyr) is the only substrate of the TCA cycle. Pyruvate is decarboxylated by pyruvate dehydrogenase (PDH) to acetyl-CoA (ACoA), which is then condensed with oxaloacetate (OA) to citrate (Cit) via the citrate synthase (CS). Citrate is converted to isocitrate (IsoCit) by the aconitase (AC), which is further converted to *α*-ketoglutarate (aKG) via the isocitrate dehydrogenase (IDH) producing NADH from NAD in the process. The *α*-ketogluterate dehydrogenase complex (KGDHC) catalyses the reaction of *α*-ketogluterate with Coenzyme A to succinyl-CoA (SucCoA) under reduction of NAD to NADH. Succinyl-CoA is further metabolized by succinyl-CoA synthase (SCS) to succinate (Suc) by phosphorylating ADP to ATP (substrate-chain phosphorylation). Succinate is dehydrogenated to fumarate (Fum) by the succinate dehydrogenase (SDH, complex II) reducing ubiquinone to ubiquinol (see legend of Figure 2). Fumerase (FUM) converts fumerate to malate (Mal), which is oxidized by malate dehydrogenase (MDH) again producing one NADH and regenerating the initial oxalacetate so the cycle can start over again. In summary, PDH and the TCA cycle produce one ATP from ADP, one ubiquinol from ubiquinone, and four NADHs from NAD while oxidizing one pyruvate to three CO_2_. Oxidation of NADH and/or succinate in the respiratory chain, is coupled to transmembrane proton pumping which generates a proton gradient and a mitochondrial membrane potential. The proton gradient is used to fuel pyruvate uptake from the cytosol into the matrix via pyruvate transporter, pumping of sodium, potassium from the matrix into the intermembrane space/cytosol, phosphate transport from the cytosol into the matrix space, and ATP generation by the F0F1-ATPase. The mitochondrial membrane potential drives the ATP/ADP exchange between the matrix and the intermembrane space/cytosol. The model also comprises the passive exchange of protons, sodium, potassium and chloride between the matrix and the intermembrane space/cytosol driven by electrodiffusion as well as the mitochondrial membrane potential. Cytosolic ATP is hydrolyzed to ADP and phosphate to meet the energy demand of the cell.

The reaction scheme of the kinetic model is shown in Figure 1. It comprises the reactions of the citric acid cycle, the respiratory chain, oxidative phosphorylation, mitochondrial ATP generation, the exchange of adenine nucleotides exchange between mitochondrial matrix and cytosol, and the transport of small ions across the inner mitochondrial membrane. Since more than 90 percent of the ATP produced in neuronal cells is derived from oxidative phosphorylation, we omitted the glycolytic pathway while putting the supply of pyruvate and its uptake into the mitochondrial matrix to a fixed value.

The kinetic model focuses on the mitochondrial-derived ATP production and is compartmentalized into cytosol and mitochondria. The model consists of 184 state variables describing the neuronal citric acid cycle, the respiratory chain, the oxidative phosphorylation, the mitochondrial ATP generation, the nucleotide exchange between the mitochondrial matrix and the cytosol, and the electrophysiological coupling between the pathways. Since more than 90 percent of the ATP produced in neuronal cells is derived from oxidative phosphorylation [16], we omitted the glycolytic pathway. It is assumed that the glycolytic pathway is not limiting in the provision of pyruvate, so cytosolic pyruvate was kept constant. Since cytosolic redox equivalents are not considered, the aspartate/malate shuttle is not part of the model. Mitochondrial calcium is a potent activator of PDH, IDH, and KGDHC. Mitochondrial calcium concentration does vary in a physiological range depending on mitochondrial membrane potential [17, 18]. For simplicity, we modeled the mitochondrial calcium concentration as linear function of the mitochondrial membrane potential.

**Figure 2:**
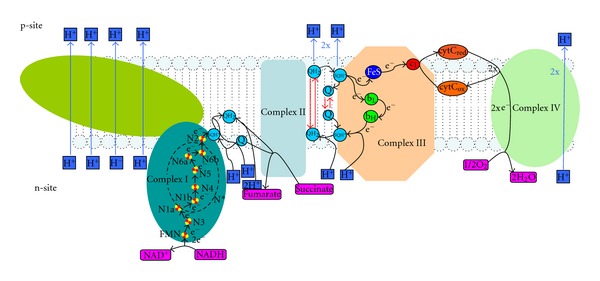
Schematic of the respiratory chain. The respiratory chain: in complex I, NADH is oxidized to NAD, while four protons are pumped from the mitochondrial matrix into the intermembrane space/cytosol. Concomitantly ubiquinon (Q), residing in the inner membranous space, is reduced to ubiquinol (QH_2_) along with the uptake of two matrix protons. In Complex II, succinate is oxidized to fumarate while ubiquinon is reduced to ubiquinol. In this reduction two protons are taken up from the matrix space, but no protons are pumped across the mitochondrial membrane. In complex III, innermembranous ubiquinol is oxidized to ubiquinon. Via the q-cycle mechanism, two protons are taken up from the matrix space, and four protons are released into the inter membrane space/cytosol. The two electrons are consecutively transferred via Fe-S cluster to cytochrome c1 and reduce two molecules of cytochrome c. In complex IV, two molecules of reduced cytochrome c are oxidized, and oxygen is reduced to water along with the transduction of two protons from the matrix space into the inter membrane space/cytosol. With either NADH or succinate as substrates, the respiratory chain pumps ten and six protons, respectively, from the matrix space to the inter membrane space/cytosol, and one molecule of water is formed.

In order to include the potential formation of ROS in our model, we developed a detailed submodel of the respiratory chain that takes into account the substructure of complexes I and III composed of several prosthetic groups and iron sulfur clusters (see Figure 2).

The functional parts of complex I are the flavine mononucleotide (FMN), eight iron-sulfur clusters (i.e., N3, N1a, N1b, N4, N5, N6a, N6b, and N2), and the docking site for ubiquinone. FMN accepts two electrons from NADH forming fully reduced flavine. The electrons are then successively transferred to the subsequent iron-sulfur clusters. When the first electron is moved, the flavin exists as a flavin radical. From the terminal iron-sulfur cluster N2, the electron is transferred to ubiquinone forming a bound semiubiquinone (SQ). A second electron transported from N2 to SQ generates ubiquinol that is released from complex I. This electron transfer takes place in the arm of complex I extending into the mitochondrial matrix, whereas the ubiquinone/ubiquinol conversion is located at the n-site of the inner mitochondria membrane. The transfer of one electron from N6b to N2 and from N2 to ubiquinone or semiubiquinone is linked with the export of one proton from the matrix into the cytosol. In the model, the electron state of complex I is represented as an array of integer numbers where the reduction of the flavinmononucleotide, each iron-sulfur cluster, and the existence of the bound semiubiquinone are encoded. All reactions are modeled as reversible mass action kinetics. Model simulations show that lumping together the complexes N1b, N4, N5, N6a, and N6b gives similar results as the full model but reduces the number of state variables from 1536 to 96.

Each state of complex III is described as an array describing the reduction and binding states of its functional parts: cytochrome c1 (c1), the iron sulfur cluster (Fe-S), the binding site for ubisemiquinone at p-site (SQ_p_), the low b-type heme (b_L_), the high b-type heme (b_H_), and the binding site for ubisemiquinone at n-site (SQ_n_). The spatial arrangement of these redox carriers enables the transduction of two electrons from one molecule ubiquinol to two molecules of cytochrome c via the q-cycle mechanism. Ubiquinol diffuses from n- to p-site, reacts with oxidized Fe-S, thereby reducing it, and generates a bound semi-ubiquinon at p-site. The bound semi-ubiquinon reduces b_L_ at p-site and free ubiquinon diffuses back to n-site. Only after release of ubiquinone from p-site, reduced Fe-S is able to transfer its electron to oxidized cytochrome c1, which passes it on to cytochrome c. The reduced b_L  _transfers its electron to oxidized b_H_. The reduced b_H_ reacts with n-site ubiquinone to bound semi-ubiquinon. In a second round, reduced b_H_ transfers its electron to bound semi-ubiquinon. In this way one electron is recycled in each turnaround of ubiquinol, and two protons are transferred from the mitochondrial matrix to the intermembrane space/cytosol per electron transferred to cytochrome c. Importantly, it is assumed that electrons are transferred from ubiquinol at p-side in a two-step process, first reducing the Fe-S and binding of SQ_p_ and afterward reducing b_L_, and release of Q_p_. As long as SQ_p_ is bound, Fe-S cannot transfer the electron to c1. This gives 48 state variable and 88 reactions for complex III. All electron transitions in complexes I and III are modeled as reversible mass action kinetics. For further details, see supplemental information in Supplementary Material available online at doi:10.1155/2012/757594.

While kinetic models of the citric acid cycle are available for heart and liver mitochondria [19], we developed a model for brain tissue. Our submodel of the respiratory chain extends existing models for complex III [20] by inclusion of cytochrome c1 and SQ_n_ into the state space and detailed modeling of complex I.

The detailed kinetic equations for the reactions and transporters (see supplemental information) are specific for neuronal and brain tissue and based on extensive literature research. V_max⁡_ values for the reactions were determined by fitting simulated metabolite concentrations to experimentally determined values.

The model was implemented in MATLAB (the MathWorks, version R2011b). The extended model of complex I of the respiratory chain was implemented in C++ (Microsoft Visual C++ 2008 Express Edition) and integrated with help of the ODE integration package CVODE (SUNDIALS). The developed source code can be provided on request.

## 3. Results

### 3.1. Analysis of Normal Mitochondrial Energy Metabolism

**Figure 3:**
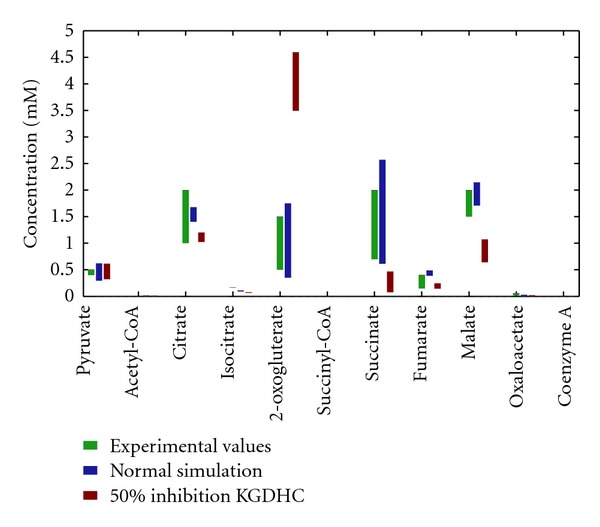
Comparison of simulated and experimentally determined concentrations of TCAC intermediates. Green bars indicate the concentration range of reported experimental values [21–29]. Blue bars (normal state) and red bars (50% inhibition of KGDHC) indicate variations of concentration when varying the energetic load between 33% and 100% of maximum.

First, we defined a normal reference state where the cytosolic ATP consumption rate, which is equal to the mitochondrial ATP production rate under steady-state conditions, amounts to about 30% of the maximal consumption rate and where in concordance with experimental data [30–32] 24% of the proton gradient is utilized by the proton leak of the inner mitochondrial membrane, 16% by pumping of potassium ions and 60% by the FoF1-ATPase and phosphate uptake. Next, we calibrated our model (unknown V_max⁡_ values) such that measured intramitochondrial metabolite concentrations (Figure 3, green bars) were reproduced. We then varied the ATP consumption rate up to its maximal possible value and calculated steady-state metabolite concentrations (Figure 3, blue bars).

**Figure 4:**
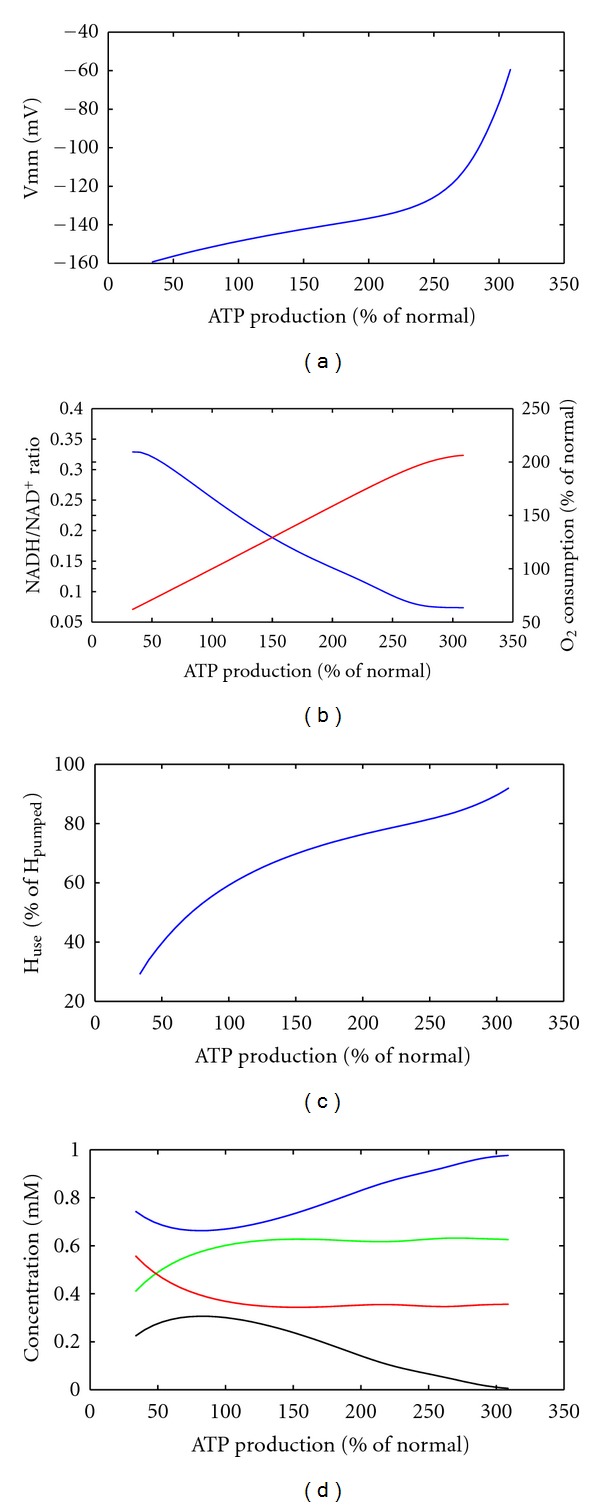
System characteristics under energetic challenge. Energetic demand was varied and behaviour of system variables determined. (a) mitochondrial membrane potential; (b) blue NADH to NAD ratio, red oxygen consumption rate; (c) share of created proton gradient used for ATP synthesis; (d) blue: ubiquinon at p-site, green: ubiquinol at n-site, red: ubiquinon at n-site, black: ubiquinol at p-site. ATP production and oxygen consumption are normalized to the reference state of the system.

To check the reliability of our model, we compared load-dependent changes of further model parameters with experimental observation reported for various tissues (Figure 4). The membrane potential is remarkably stable between −150 mV and −120 mV over a wide range of ATP consumption rates [33]. However, beyond a 2.5-fold increase of the ATP consumption rate, a small further increase in the ATP consumption rate was accompanied by a large drop in the membrane potential, hinting to metabolic failure. The mitochondrial redox potential (expressed through the NADH/NAD ratio) showed a quasilinear decline from 0.3 to 0.1 for ATP consumption rates up to the 2.5-fold of the normal. Concurrently, the oxygen consumption rate doubled. The fact that oxygen consumption only doubled at threefold increased ATP consumption is accounted for by increased efficiency of ATP production, that is, the share of ATP generation in the utilization of the proton gradient increases from initially 60% to over 90% (Figure 4(c)). The total ubiquinol to ubiquinon ratio (at n-site + p-site) varied between 1.5 and about 0.5, in agreement with experimental data [29]. At the mitochondrial p-site, this ratio dropped from about 1 to zero at maximal indicating that ubiquinol diffusion becomes rate limiting at high ATP consumption rates [34].

**Figure 5:**
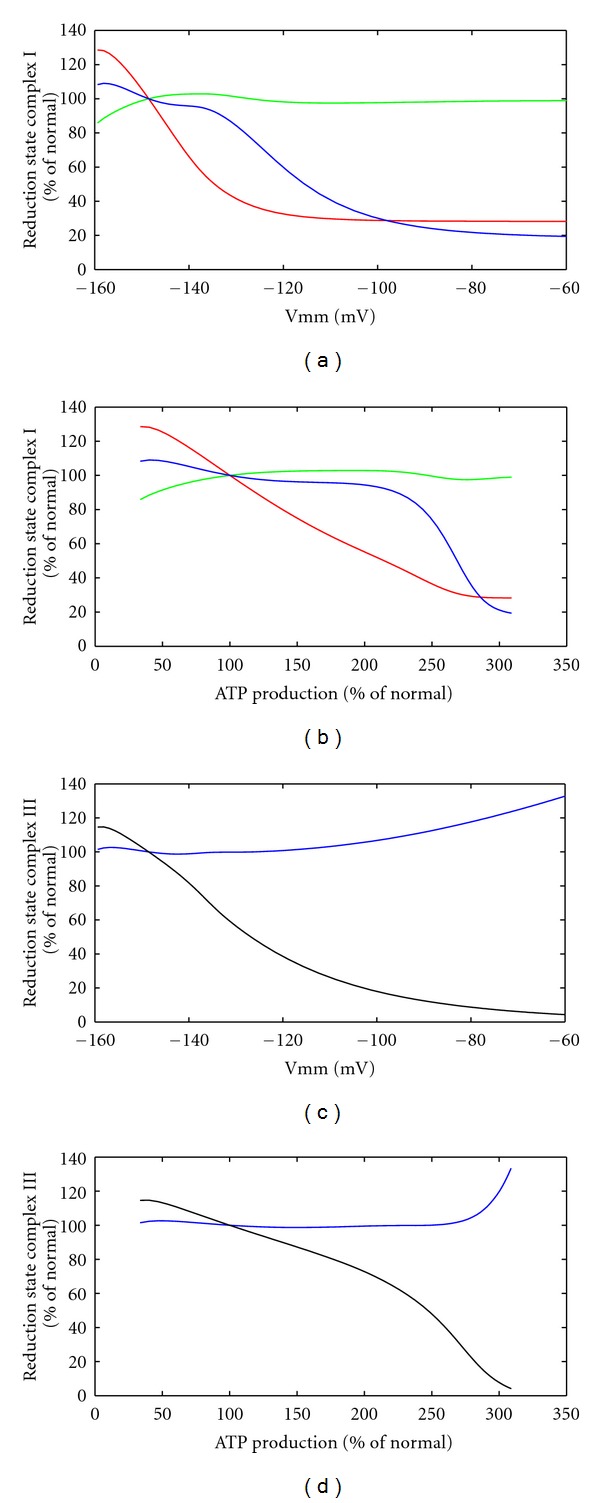
Potential ROS producing states in the RC. ROS producing states of complex I ((a) and (b)) and complex III ((c) and (d)) are depicted versus the mitochondrial membrane potential ((a) and (c)) or the ATP production rate. Red: fully reduced flavin, green: flavin radical, blue: semi-ubiquinon at n-site bound to the respective complex, black: semi-ubiquinon at p-site bound to complex III. ATP production and occupation of ROS producing states are normalized to the reference state of the system.

Since multiple sites for mitochondrial ROS production in complex I and complex III have been suggested in the literature, we monitored the occupation states of the disputed ROS producing sites at varying ATP consumption rate (see Figure 5). From our model simulations, we concluded that the fully reduced flavin, the semi-ubiquinone bound at n-site in complex I, and the semiubiquinone bound a t p-site in complex III are in agreement with expected dependencies on the membrane potential, while the flavin radical and the semiubiquinone at p-site can be ruled out as main ROS producers.

### 3.2. Analysis of Mitochondrial Energy Metabolism at Reduced KGDHC Activities

**Figure 6:**
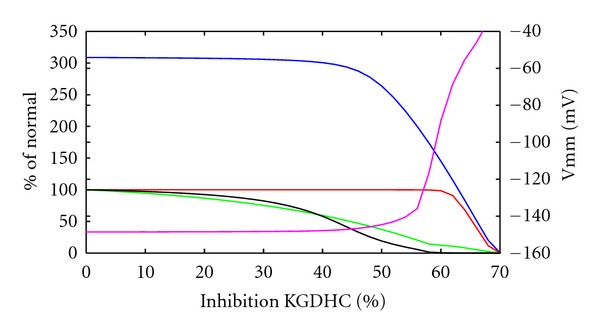
System characteristics under KGDHC inhibition. ATP production rate (red), NADH level (green), mitochondrial membrane potential (magenta, right scale), and reduced cytochrome c level (black) at normal ATP demand versus increased inhibition of KGDHC, maximal ATP production capacity in blue. Values except membrane potential normalized to reference state without inhibition.

Next, we investigated the effects of KGDHC and aconitase inhibition on the energy metabolism. Simulations were performed with increasing degree of inhibition of the KGDHC from 0 to 70% (see Figure 6). The maximal ATP production capacity decreased very slowly until about 50% inhibition of the enzyme. Higher inhibition resulted at first in an approximately linear decrease of the maximal ATP production capacity and finally also in a linear reduction of the ATP production rate in the reference state, that is, the energy demand of the normal load state can be satisfied until about 60% inhibition. At an inhibition of about 70%, the system is close to collapse as can be seen by the highly depolarized membrane potential. Successive depletion of NADH (green curve) caused depletion of reduced cytochrome c (black curve) in a nonlinear manner which is ultimately responsible for the metabolic failure seen at high inhibition states. Notably, as long as reduced cytochrome c is not fully exhausted, the membrane potential can be kept close to the reference value. Caution must be used at interpreting the curves in regimes with strongly depolarized mitochondrial membrane (>−80 mV), since mechanisms not modeled (like initiation of apoptotic pathway/transition pore opening) are likely to dominate the cellular behavior.

There occurred also profound shifts in the metabolite concentrations of the citric acid cycle intermediates. Figure 3 shows the range of the citric acid cycle intermediates under varied energetic load conditions at 50% inhibition of the KGDHC (red bars). A-ketoglutarate was vastly increased and thus partially compensated for the loss in enzyme activity through higher substrate availability. Other metabolites of the TCAC had lower concentrations compared to the normal case.

**Figure 7:**
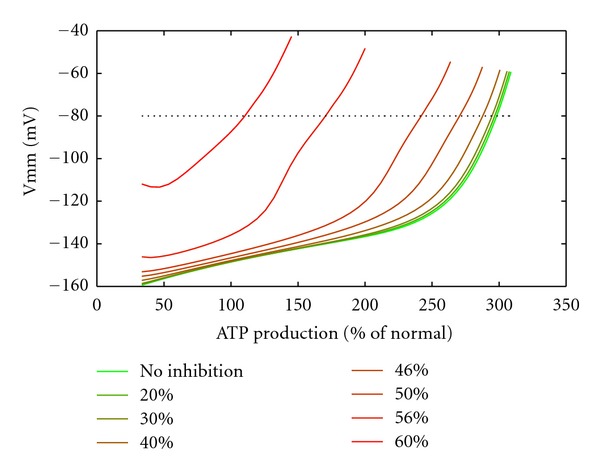
Mitochondrial membrane potential characteristics at inhibition of KGDHC. Mitochondrial membrane potential versus the ATP production rate at different inhibition levels of KGDHC. Dotted line: −80 mV membrane potential level.

The dependence of the mitochondrial membrane potential on the ATP consumption rate at different inhibitions of the KGDHC is shown in Figure 7. At moderate inhibition of the KGDHC <30%, the increase of the membrane potential was only 20 mV up to 2.5-fold elevated energetic load, that is, a significant rise of the membrane potential occurred only at very high energetic load. At KGDHC inhibition of about 60%, the membrane potential was already elevated at normal ATP consumption rate, and the membrane depolarized at much smaller increase of the ATP demand. Given pathological states of mitochondria to occur at values of the membrane potential above −80 mV (dotted line in Figure 7), increasing KGDHC inhibition of the membrane potential resulted in a dramatic reduction of the tolerable maximal energetic load and successive membrane depolarization at normal energy demand.

**Figure 8:**
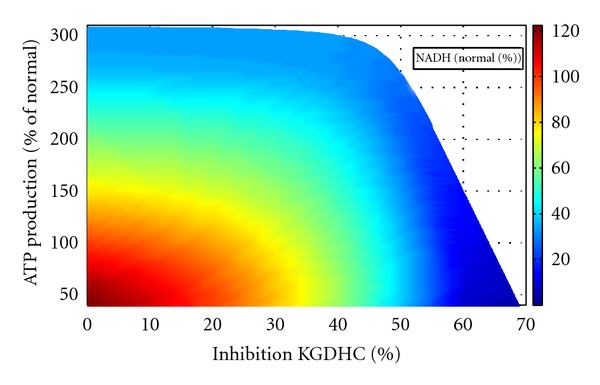
NADH level characteristics at varying ATP demand and KGDHC inhibition. NADH level is depicted as colour value (right scale). NADH level and ATP production are normalized to the reference state without KGDHC inhibition.

**Figure 9:**
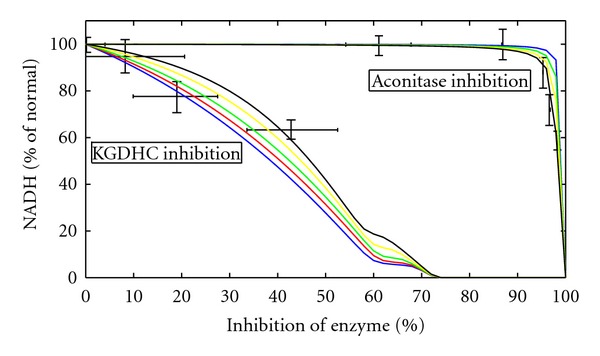
NADH level comparison experiment and simulation. Experimental determined NADH level as black points with error bars at different inhibition levels of KGDHC and aconitase (data from [15]). Different coloured curves vary in the basal ATP demand of the system from low (blue) to increased (black). Green curves are for the ATP demand of the reference state. NADH levels are normalized to the NADH level without enzyme inhibition respective to the ATP demand.

The redox state of the RC and thus the residual energetic capacity of the mitochondrion is common level.  Figure 8 shows the combined impact of KGDHC inhibition and energetic load on the mitochondrial NADH level. Generally, progressive inhibition of the KGDHC is equivalent to progressive increase of energetic load. For example, relative inhibition of about 40% has the same effect as a 1.5-fold increase of the energetic load by the mitochondrial NADH. The effect of KGDHC and aconitase inhibition on mitochondrial NADH content has been determined experimentally [15].  Figure 9 demonstrates that our simulations are in good concordance with these experimental data. Inhibition of the KGDHC has a much stronger effect on the NADH content than inhibition of the aconitase. Whereas half-reduction of the NADH level is already achieved with about 40% inhibition of the KGDHC, the same effect requires about 95% inhibition of the aconitase.

### 3.3. ROS Production in the Respiratory Chain at Inhibited KGDHC

**Figure 10:**
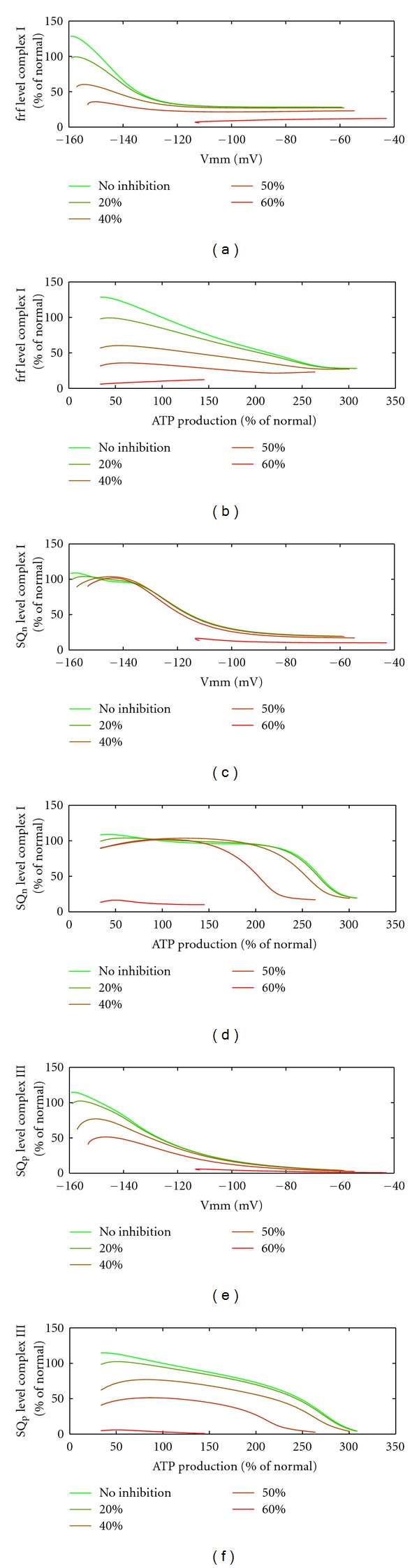
ROS production at inhibition of KGDHC. Levels of fully reduced flavin ((a) and (b)), semi-ubiquinone at n-site bound to complex I ((c) and (d)) and semi-ubiquinone at p-site bound to complex III ((e) and (f)) at various inhibitions (a) of KGDHC. The levels are depicted versus the mitochondrial membrane potential ((a), (c), and (e)) and the ATP production rate ((b), (d) and (f)). Values except membrane potential are normalized to the reference state.

Next, we investigated the influence of KGDHC inhibition on ROS generation by the RC. To this end, we calculated the occupation state of ROS generating sites in the presence of KGDHC inhibition. Since our simulation of the normal case suggested that the flavin radical in complex I and the semiubiquinone at n-site in complex III can be discarded as major ROS generating sites, only the fully reduced flavin, the semiubiquinone bound at n-site of complex I, and the semiubiquinone bound at p-site of complex III are shown. With increasing degree of KGDHC inhibition, there was a remarkable reduction in the occupation state of the fully reduced flavin in complex I as well as SQ_p_ of complex III at all workloads (see Figure 10), while the changes of SQ_n_ of complex I were negligibly small. Above 2.5-fold increase of the energetic load corresponding to a rise of the membrane potential above −100 mV, the RC is almost completely oxidized so that additional KGDHC inhibition has only a marginal effect on the occupation state of the considered ROS generating sites.

## 4. Discussion

Decreased KGDHC activity in neuronal cells of the brain is associated with a number of neurodegenerative diseases. To understand the impact of reduced KGDHC on the mitochondrial energy metabolism of neurons, we developed a detailed mathematical model comprising the central components of mitochondrial ATP generation.

In agreement with experimental findings, our model simulations showed a steady decline of the mitochondrial NADH level with progressive KGDHC inhibition, whereas up to 95% inhibition of the aconitase had virtually no impact. This finding underpins the notion of KGDHC to be a rate-limiting enzyme of the TCAC. Decline of NADH fluorescence at inhibition of KGDHC has been considered to be indicative for a reduced ATP-generating capacity of mitochondria [15]. The advantage of our mathematical model is to enable predictions of the relationship between NADH decline and ATP production rate. These simulations suggest that a reduced NADH content does not translate linearly to reduced energy production. This is due to a compensatory change in the level of TCAC intermediates upon KGDHC inhibition, thus rendering the flux through the TCAC almost constant over a wide range of inhibition at normal or moderately enhanced energetic workload. Increased akg levels activate the remaining intact KGDHC to reestablish the original flux through the enzyme. This leads to an increased akg content (see Figure 3), a marker for KGDHC impairment, since akg levels are also elevated in the blood/urine. Since akg is in equilibrium with glutamate by a deaminase reaction, this might lead to glutamate poisoning, another pathological feature associate with KGDHC inhibition [35].

Our simulation also revealed that the impairment of the energy metabolism depends on the functional state of the neuron. At high inhibition and/or high energetic workload, the energetic output is severely compromised, and functionality may not be maintained. This goes along with a marked membrane depolarization.

There is striking evidence for a correlation between reduced KGDHC activity and the loss of glutamatergic neurons seen for example, in Alzheimer's and Parkinson's disease [11]. Programmed cell death through the apoptotic pathway requires the simultaneous occurrence of three different events: depolarization of the mitochondrial membrane potential, low intramitochondrial ATP concentrations, and a mitochondrial overload with calcium. The first two conditions are fulfilled when neurons with reduced KGDHC activity are challenged with high ATP demand that normally can be accomplished by the cell. When the cytosolic ATP levels are low, calcium is not sufficiently pumped out of the cell into the extracellular space, and the resulting increased cytosolic calcium concentration finally leads to mitochondrial calcium overload. Thus, strongly reduced KGHDC activity might directly lead to apoptosis.

One of the peculiarities of neurodegenerative diseases is the strong age dependence. It is believed that the accumulation of mitochondrial damage through ROS is one of the determining factors in brain aging and performance as well as a key factor in the development of neurodegenerative diseases [36]. One of the harmful effects of ROS is the inactivation of enzymes of the citric acid cycle. Citric acid cycle enzymes that are especially susceptible to oxidative stress are the aconitase and the KGDHC [15, 37]. Aconitase shows the highest susceptibility towards ROS, because of its sulfur-iron complex, but aconitase inhibition remains irrelevant if it does not exceed 90 percent (see Figure 9 and [15]). KGDHC is tightly bound to the inner mitochondrial membrane [38] and might be part of a citric acid cycle super-complex [39]. It binds to complex I of the mitochondrial respiratory chain [40], which might make it a prominent target of ROS due to the close spatial proximity to the ROS generating sites. Thus, ROS is one possible reason for KGDHC deficiency.

There is no general agreement on the relative importance of various proposed ROS producing sites of the RC. Dependence of ROS production from the membrane potential has been measured [41, 42]. Comparing these measured characteristics with the occupation status of the disputed ROS generating sites, modeling can help to identify or exclude sites as relevant producers of ROS (see Figure 5). Our calculations show that the occupation state as function of the membrane potential of the fully reduced flavin, the SQ_n_ site in complex I, and the SQ_p_ site in complex III, but not the flavin radical in complex I, and the SQ_n_ site in complex III, is in agreement with measured dependencies. For complex I, our findings are in agreement with experimental results in [43] excluding the flavin radical but showing the fully reduced flavin to act as ROS producing site of complex I. For complex III, our findings are in agreement with other reports (see, e.g. [44]).

Since KGDHC is especially susceptible to ROS, we examined if the reduction of KGDHC activity has an effect on the occupation state of the confirmed relevant ROS producing sites (Figure 10). We found that there is a significant decrease in the occupation state of the fully reduced flavin in complex I and SQ_p_ of complex III for all ATP demands, that is, KGDHC inhibition reduces ROS production in the respiratory chain by lowering the redox state of the mitochondrion. Since KGDHC itself is also an ROS producer and ROS production by this enzyme decreases with decreasing mitochondrial redox state [45], endogenous ROS production by KGHC is also reduced in case of KGDHC deficiency.

It has to be noted that our model does not include the impairment of RC complexes by ROS as it has been observed in neurodegenerative diseases (see, e.g., [46]). A reduction in the activity of complex I results in increase in ROS production [47]. Hence, structural and functional impairment of the RC might override the inhibitory effect of reduced KGDHC on ROS production.

Here, we focused on the effects of KGDHC inhibition on the energy metabolism and showed that for a given degree of inhibition the impairment depends crucially on the functional state of the cell. For ROS production, KGDHC inhibition alone does reduce the ROS generation in the respiratory chain. Nevertheless, the detailed description of the RC in our model provided further arguments for the relative importance of ROS producing sites proposed in the literature.

Taken together, our model helps to elucidate the causal chain of molecular events connecting reduced KGDHC activity to the energetic breakdown of neuronal cells during development of neurodegenerative diseases.

## Supplementary Material

The supplementary material contains the detailed kinetics of the model. It is subdivided into Oxidative phosphorylation, Electrophysiology containing the respiratory chain, and the citric acid cycle (CAC).

## Abbreviations

Fe-S: Iron sulfur
FMN: Flavin mononucleotide
KGDHC: *α*-ketoglutarate dehydrogenase
RC: Respiratory chain
ROS: Reactive oxygen species
SQ: Semi-ubiquinon
TCAC: Tricarbonic acid cycle/citric acid cycle
Q: Ubiquinon
QH_2_: Ubiquinol.
