# Corrigendum to “Kinetic Modeling of the Mitochondrial Energy Metabolism of Neuronal Cells: The Impact of Reduced α-Ketoglutarate Dehydrogenase Activities on ATP Production and Generation of Reactive Oxygen Species”

**DOI:** 10.1155/2018/6139262

**Published:** 2018-09-09

**Authors:** Nikolaus Berndt, Sascha Bulik, Hermann-Georg Holzhütter

**Affiliations:** Institute of Biochemistry, University Medicine-Charité, 13347 Berlin, Germany

In the article titled “Kinetic Modeling of the Mitochondrial Energy Metabolism of Neuronal Cells: The Impact of Reduced *α*-Ketoglutarate Dehydrogenase Activities on ATP Production and Generation of Reactive Oxygen Species” [1], there was an error in Figure 1, in which the decarboxylation of pyruvate to acetyl-CoA should be accompanied by NAD to NADH, not NADH to NAD. The figure should be corrected as in Figure 1.

## Figures and Tables

**Figure 1:**
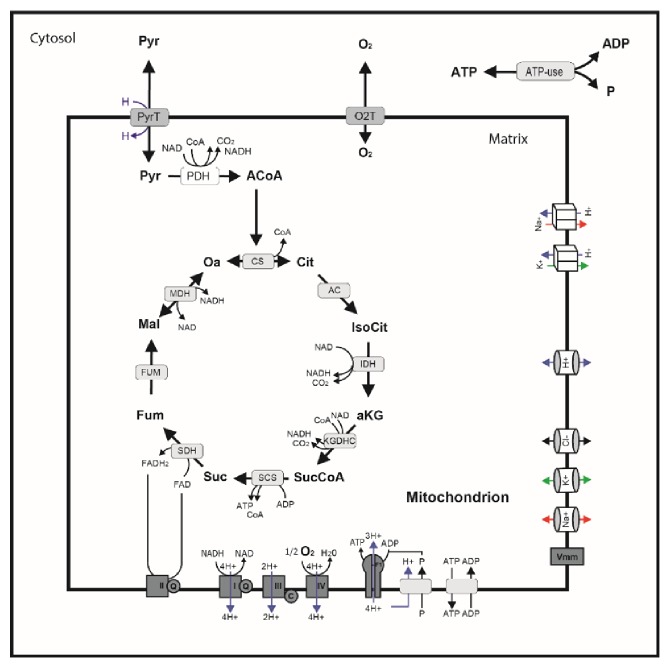
Schematic of the mathematical model. Pyruvate is the only substrate of the TCA cycle. Pyruvate is decarboxylated by pyruvate dehydrogenase (PDH) to acetyl-CoA, which is then condensed with oxalacetate to citrate via the citrate synthase (CS). Citrate is converted into isocitrate by the aconitase (AC), which is further converted to *α*-ketoglutarate via the isocitrate dehydrogenase (IDH) producing NADH from NAD in the process. The *α*-ketoglutarate dehydrogenase complex (KGDHC) catalyzes the reaction of *α*-ketoglutarate with Coenzym A to succinyl-CoA under reduction of NAD to NADH. Succinyl-CoA is further metabolized by succinyl-CoA synthase (SCS) to succinate by phosphorylating ADP to ATP (substrate-chain phosphorylation). Succinate is dehydrogenated to fumarate by the succinate dehydrogenase (SDH, complex II) reducing ubiquinone to ubiquinol (see legend, Figure 2). Fumarase (FUM) converts fumarate into malate, which is oxidized by malate dehydrogenase (MDH) again producing one NADH and regenerating the initial oxalacetate so the cycle can start over again. In summary, PDH and the TCA cycle produces one ATP from ADP, one ubiquinol from ubiquinone, and four NADH from NAD while oxidizing one pyruvate to three CO_2_. Oxidation of NADH and/or succinate in the respiratory chain is coupled to transmembrane proton pumping which generates a proton gradient and a mitochondrial membrane potential. The proton gradient is used to fuel pyruvate uptake from the cytosol into the matrix via pyruvate transporter, pumping of sodium, potassium from the matrix into the intermembrane space /cytosol, phosphate transport from the cytosol into the matrix space, and ATP generation by the F0F1-ATPase. The mitochondrial membrane potential drives the ATP/ADP exchang**e** between the matrix and the intermembrane space /cytosol. The model also comprises the passive exchange of protons, sodium, potassium, and chloride between the matrix and the intermembrane space/cytosol driven by electro diffusion as well as the mitochondrial membrane potential. Cytosolic ATP is hydrolyzed to ADP and phosphate to meet the energy demand of the cell.
