# Supplementary material for: Kinetic Modeling of the Mitochondrial Energy Metabolism of Neuronal Cells: The Impact of Reduced α-Ketoglutarate Dehydrogenase Activities on ATP Production and Generation of Reactive Oxygen Species
Source: Int J Cell Biol. 2012 Jun 10;2012:757594. doi: 10.1155/2012/757594 (PMC3376505; doi:10.1155/2012/757594)
Supplement: Supplementary file 1 — The supplementary material contains the detailed kinetics of the model. It is subdivided into Oxidative phosphorylation, Electrophysiology containing the respiratory chain, and the citric acid cycle (CAC). [file 757594.f1.pdf]

Oxidative phosphorylation:

ATP-Synthetase [1, 2]

(ATP -> ADP + Pi)

$$v_{syn} = V_{\max}^{syn} \cdot \left(ADP_{mito} \cdot P_{mito} - \frac{ATP_{mito}}{K_{eq-syn}} \right)$$

$$K_{eq-syn} = \exp\left(\frac{dG_0^{syn}}{R \cdot T} - k \cdot U \right) \cdot \left(\frac{h_{cyt}}{h_{mito}} \right)^k$$

$$dG_0^{syn} = 30500$$

$$k = 3$$

ATP-exchanger: [3]
(ATP_mito + ADP_cyt <-> ADP_mito + ATP_cyt)

$$v_{ATP-exchanger} = V_{\max}^{ATP-exchanger} \left(\frac{1 - \frac{ATP_{in} \cdot ADP_{mito}}{ADP_{in} \cdot ATP_{mito}} \exp(U)}{\left(1 + \frac{ATP_{in}}{ADP_{in}} \exp\left(S_{V_{mm}} \cdot U \right) \right) \left(1 + \frac{ADP_{mito}}{ATP_{mito}} \right)} \right)$$

$$S_{V_{mm}} = 0.3$$

ATP-consumption

ATP consumption is modeled as a Michaelis-Menten equation with low K_m-value:

$$v_{ATP-use} = V_{\max-ATP-use} \frac{ATP_{in}}{ATP_{in} + K_m^{ATP}}$$

$$K_m^{ATP} = 0.1$$

Electrophysiology:

$$U = \frac{V_{mm} \cdot F}{1000 \cdot R \cdot T}$$

$$F = 96490.0 \frac{C}{mol}$$

$$R = 8.314 \frac{J}{K \cdot mol}$$

$$T = 310 \ K$$

Electro diffusion:

The passive efflux of sodium ions, potassium ions, chloride ions and protons is modeled by the Goldman-Hodgkin equation.

Potassium [4]

$$I_{Pot_{mito}}^{ed} = A_m \cdot P_{Pot_{mito}} \cdot U \cdot F \cdot \left(\frac{K_{in} - K_{mito} \cdot \exp(U)}{1 - \exp(U)} \right)$$

$$P_{Pot_{mito}} = 2 \cdot 10^{-10} \frac{m}{s}$$

Sodium

$$I_{Na\,mito}^{ed} = A_m \cdot P_{Na_{mito}} \cdot U \cdot F \cdot \left(\frac{Na_{in} - Na_{mito} \cdot \exp(U)}{1 - \exp(U)} \right)$$

$$P_{Na_{mito}} = 1 \cdot 10^{-11} \frac{m}{s}$$

Protons: [5], [6]

$$I_{H_{mito}}^{ed} = -A_m \cdot P_{H_{mito}} \cdot U \cdot F \cdot \left(\frac{H_{in} - H_{mito} \cdot \exp(U)}{1 - \exp(U)} \right)$$

$$P_{H_{mito}} = 4 \cdot 10^{-4} \frac{m}{s}$$

Pumps: [7]

The pumping of sodium, potassium and phosphate is modeled as electro-neutral proton driven antiport:

$$v_{Phos-exchanger} = V_{\max}^{Phos-exchanger} \left(P_{in} \cdot H_{in} - P_{mito} \cdot H_{mito} \right)$$

$$I_{Na_{mito}}^{pump} = V_{\max}^{Na-pump} \left(Na_{in} \cdot H_{mito} - Na_{mito} \cdot H_{in} \right)$$

$$I_{Pot_{mito}}^{pump} = V_{\max}^{Pot-pump} \left(K_{in} \cdot H_{mito} - K_{mito} \cdot H_{in} \right)$$

Complex III: [8], [9], [10]

The state of complex III is described as an array consisting of two numbers, a letter, two numbers and another letter. The numbers denote the reduction state, 0 for oxidized, 1 for reduced, of the corresponding complex III elements, the letter the binding, b, or not binding, n, of the ubisemiquinone. From left to right the array position denotes the reduction state of the cytochrome c1 (c1), the reduction state of the Iron sulfur cluster (Fe-S), the binding of the ubisemiquinone at p site (SQ_p), the reduction state of the low b-type heme (b_L), the reduction state of the high b-type heme (b_H) and the binding of the ubiseiquinone at n site (SQ_n).

$$\begin{matrix} c1 & Fe-S & SQ_p & b_L & b_H & SQ_n \\ 0/1 & 0/1 & n/b & 0/1 & 0/1 & n/b \end{matrix}$$

As examples 00n00n describes the fully oxidized complex III, with no bound ubisemiquinone, while 11b11b describes the fully reduced complex III with ubisemiquinone bound at n and p site, and 10n11b describes the state of complex III with reduced cytochrome c1, oxidized Fe-S, reduced b-type heme b_L, reduced b-type heme b_H and bound ubisemiquinone at n but not at p side.

There are seven different reversible elementary processes with rate equation:

- **P site ubiquinol reacts with oxidized Fe-S to reduced Fe-S and p-site bound ubisemiquinone and release of two protons into the cytosol**

  $(QH_2)_p$+ x0nxxx <-> x1bxxx + h_{in}

$$v_{SQ_p} = V_{\max}^{SQ_p}\left((QH_2)_p \cdot x0nxxx - \frac{1}{K_{eq}^{SQ_p}} \cdot x1bxxx \right)$$

$$K_{eq}^{SQ_p} = \exp\left(\frac{f_V^{SQ_p} \cdot V \cdot F}{R \cdot T \cdot 1000} \right) \cdot \frac{h_{cyt}}{h_{ref}}$$

$$f_V^{SQ_p} = 0.1$$

$$h_{ref} = 10^{-7}$$

- **Reduction of the low b-type heme and release of ubiquinon at p site:**
  x1b0xx <-> x1n1xx + Q_p + h_{in}

$$v_{Q_p} = V_{\max}^{Q_p}\left(x1b0xx - \frac{1}{K_{eq}^{Q_p}} \cdot x1n1xx \cdot Q_p \right)$$

$$K_{eq}^{Q_p} = \exp\left(\frac{\left(E_0^{Q_p} + f_V^{Q_p} \cdot V\right) \cdot F}{R \cdot T \cdot 1000} \right) \cdot \frac{h_{cyt}}{h_{ref}}$$

$$f_V^{Q_p} = 0.1$$

$$E_0^{Q_p} = 120$$

$$h_{ref} = 10^{-7}$$

- **Electrontransfer between the low b–type heme b_L and the high b-typ heme b_H:**
  xxx10x <-> xxx01x

$$v_{hemes} = V_{\max}^{hemes}\left(xxx10x - \frac{1}{K_{eq}^{hemes}} \cdot xxx01x \right)$$

$$K_{eq}^{hemes} = \exp\left(\frac{\left(E_0^{hemes} + f_V^{hemes} \cdot V\right) \cdot F}{R \cdot T \cdot 1000} \right)$$

$$f_V^{hemes} = 0.8$$

$$E_0^{hemes} = 80$$

- **N site ubiqinone reacts with reduced high b-type heme to bound semiquinone at n site:**
  xxxx1n + h_{mito}+Q_n <->xxxx0b

$$v_{SQ_n} = V_{\max}^{SQ_n}\left(Q_n \cdot xxxx1n - \frac{1}{K_{eq}^{SQ_n}} \cdot xxxx0b \right)$$

$$K_{eq}^{SQ_n} = \exp\left(\frac{\left(E_0^{SQ_n} + f_V^{SQ_p} \cdot V\right) \cdot F}{R \cdot T \cdot 1000} \right) \cdot \frac{h_{mito}}{h_{ref}}$$

$$f_V^{SQ_n} = 0.1$$

$$E_0^{SQ_n} = 30$$

- **Reduced high b-type heme reacts with n site bound ubisemiquinone:**
  xxxx1b + h_{mito} <-> xxxx0n + $(QH_2)_n$

$$v_{(QH_2)_n} = V_{\max}^{(QH_2)_n}\left(\cdot xxxx1b - \frac{1}{K_{eq}^{(QH_2)_n}} \cdot xxx0n \cdot \left(QH_2\right)_n \right)$$

$$K_{eq}^{(QH_2)_n} = \exp\left(\frac{\left(E_0^{(QH_2)_n} + f_V^{(QH_2)_n} \cdot V\right) \cdot F}{R \cdot T \cdot 1000} \right) \cdot \frac{h_{mito}}{h_{ref}}$$

$$f_V^{(QH_2)_n} = 0.1$$

$$E_0^{(QH_2)_n} = 130$$

$$h_{ref} = 10^{-7}$$

- **Reduced Fe-S reacts with oxidized cytochrome c1 :**
  01xxxx <-> 10xxxx

$$v_{cytc1} = V_{\max}^{cytc1}\left(01xxxx - \frac{1}{K_{eq}^{cytc1}} \cdot 10xxxx \right)$$

$$K_{eq}^{cytc1} = \exp\left(\frac{E_0^{cytc1} \cdot F}{R \cdot T \cdot 1000} \right)$$

$$E_0^{cytc1} = -35$$

- **Reduced cytochrome c1 reacts with oxidized cytochrom c:**
  1xxxxx + $cytc_{ox}$ <-> 0xxxxx + $cytc_{red}$

$$v_{cytc} = V_{\max}^{cytc}\left(1xxxxx \cdot cytc_{ox} - \frac{1}{K_{eq}^{cytc1}} \cdot 0xxxxx \cdot cytc_{red} \right)$$

$$K_{eq}^{cytc} = \exp\left(\frac{E_0^{cytc} \cdot F}{R \cdot T \cdot 1000} \right)$$

$$E_0^{cytc} = -10$$

Complex I : [11], Hirst 2005 Biochemical Society Transaction vol 33, Rouslap 2010 nature
(NADH + Q + 4 H_mito <-> NAD + QH2 + 4 H_in)

Similar to complex III the state of complex I is described as an array of integer numbers of length 10, one number for the flavin, eight for the iron sulfur clusters, and one for the semi-ubiquinone. The flavin mononucleotide can be fully reduced (state=2), exist as flavin radical (state=1) or by fully oxidized (state=0). The iron-sulfur clusters of complex I can exist in oxidized form (represented by a zero) or in a reduced form (state=1). The last digit in the array represents the bound semi-ubiquinone, which can be absent (state=0) or bound (state=1).

FMN	$N3$	$N1a$	$N1b$	$N4$	$N5$	$N6a$	$N6b$	$N2$	SQ
2/1/0	1/0	1/0	1/0	1/0	1/0	1/0	1/0	1/0	1/0

This results in 1536 state variables and twelve elementary processes, generating 4480 reactions. This system of complex I was analyzed and reduced to a simplified system containing only six clusters. The iron sulfur clusters N1b, N4, N5, N6a, and N6b were lumped together to N* due to their similar midpoint potentials. The reduced system consists of 96 state variables, eight elementary processes and therewith 184 reactions. The occupation states of the remaining clusters are similar between the full and the reduced system. For the here developed model of the citric acid cycle and respiratory chain the simplified model was used.

FMN	$N3$	$N1a$	$N*$	$N2$	SQ
2/1/0	1/0	1/0	1/0	1/0	1/0

There are eight different reversible elementary processes:

- **Mitochondrial NADH reacts with oxidized FMN to NAD^+ and frf.**

  $NADH_{mito}$+ 0xxxxx <-> NAD^+ + 2xxxxx

$$v_{frf} = V_{max}^{frf}\left(NADH_{mito}\cdot 0xxxxx - \frac{1}{K_{eq}^{frf}}\cdot NAD_{mito}^{+}\cdot 2xxxxx\right)$$

$$K_{eq}^{frf} = \exp\left(\frac{E_0^{frf}\cdot F}{R\cdot T\cdot 1000}\right)$$

$$E_0^{frf} = -32mV$$

- **One electron is transferred from the fully reduced flavin mononucleotide to N3.**

  20xxxx <-> 11xxxx

$$v_{frf-N3} = V_{max}^{frf-N3}\left(20xxxx - \frac{1}{K_{eq}^{frf-N3}}\cdot 11xxxx\right)$$

$$K_{eq}^{frf-N3} = \exp\left(\frac{E_0^{frf-N3}\cdot F}{R\cdot T\cdot 1000}\right)$$

$$E_0^{frf-N3} = 12mV$$

- **Transfer from flavine-radical to N3.**

  10xxxx <-> 01xxxx

$$v_{fr-N3} = V_{max}^{fr-N3}\left(10xxxx - \frac{1}{K_{eq}^{fr-N3}}\cdot 01xxxx\right)$$

$$K_{eq}^{fr-N3} = \exp\left(\frac{E_0^{fr-N3} \cdot F}{R \cdot T \cdot 1000}\right)$$

$$E_0^{frf-N3} = 170mV$$

- **From N3 to N1A.**

x10xxx <-> x01xxx

$$v_{N3-N1a} = V_{\max}^{N3-N1a}\left(x10xxx - \frac{1}{K_{eq}^{N3-N1a}} \cdot x01xxx\right)$$

$$K_{eq}^{N3-N1a} = \exp\left(\frac{E_0^{N3-N1a} \cdot F}{R \cdot T \cdot 1000}\right)$$

$$E_0^{N3-N1a} = -135mV$$

- **From N1a to N*.**

xx10xx <-> xx01xx

$$v_{N1a-N*} = V_{\max}^{N1a-N*}\left(xx10xx - \frac{1}{K_{eq}^{N1a-N*}} \cdot xx01xx\right)$$

$$K_{eq}^{N1a-N*} = \exp\left(\frac{E_0^{N1a-N*} \cdot F}{R \cdot T \cdot 1000}\right)$$

$$E_0^{N1a-N*} = 130mV$$

- **From N* to N2, thereby exporting one proton from the mitochondrial matrix.**

xxx10x + h_{mito} <-> xxx01x + h_{in}

$$v_{N*-N2} = V_{\max}^{N*-N2}\left(xxx10x - \frac{1}{K_{eq}^{N*-N2}} \cdot xxx01x\right)$$

$$K_{eq}^{N*-N2} = \exp\left(\frac{\left(V_{mm} + E_0^{N*-N2}\right) \cdot F}{R \cdot T \cdot 1000}\right) \cdot \frac{h_{mito}}{h_{in}}$$

$$E_0^{N*-N2} = 150mV$$

$$h_{ref} = 10^{-7}$$

- **Binding of ubichinon to complex I and transfer of one electron from N2 forming bound semi-ubichinon and exporting one proton from the mitochondrial matrix.**

xxxx10 + Q + h_{mito} <-> xxxx01 + h_{in}

$$v_{N2-SQ} = V_{\max}^{N2-SQ}\left(Q \cdot xxxx10 - \frac{1}{K_{eq}^{N2-SQ}} \cdot xxxx01\right)$$

$$K_{eq}^{N2-SQ} = \exp\left(\frac{\left(V_{mm} + E_0^{N2-SQ}\right) \cdot F}{R \cdot T \cdot 1000}\right) \cdot \frac{\left(h_{mito}\right)^2}{h_{in} \cdot h_{ref}}$$

$$E_0^{N2-SQ} = 185mV$$
$$h_{ref} = 10^{-7}$$

- **Transfer of one electron from N2 to bound semi-ubichinon forming and releasing ubichinol, thereby exporting one proton from the mitochondrial matrix.**

xxxx11 + h_{mito} <-> xxxx00 + QH_2 + h_{in}

$$v_{N2-QH_2} = V_{\max}^{N2-QH_2}\left(xxxx11 - \frac{1}{K_{eq}^{N2-QH_2}} \cdot xxxx00 \cdot QH_2 \right)$$
$$K_{eq}^{N2-QH_2} = \exp\left(\frac{\left(V_{mm} + E_0^{N2-QH_2}\right)\cdot F}{R\cdot T\cdot 1000} \right)\cdot \frac{\left(h_{mito}\right)^2}{h_{in}\cdot h_{ref}}$$
$$E_0^{N2-SQ} = 225mV$$
$$h_{ref} = 10^{-7}$$

Complex IV [12], [13], [14]
(2 cyt_red + O2 + 4 H_mito -> H2O + 2 cyt_ox + 2 H_ in)

$$v_{C_4} = V_{\max}^{Cx4}\cdot \frac{cytC_{red}^{\ n}}{cytC_{red}^{\ n} + \left(K_m^{cytC_{red}}\right)^n} \frac{O_{2mito}}{O_{2mito} + K_m^{O_2}} \left(\frac{h_{mito}}{h_{in}} \right)^6$$

$$K_m^{O_2} = 0.001$$
$$n = A + B\frac{\left(\frac{ATP_{mito}}{ADP_{mito}} \right)^k}{\left(\frac{ADP_{mito}}{ADP_{mito}} \right)^k + \left(K_n^{\frac{ATP}{ADP}} \right)^k}$$
$$A = 1.1$$
$$B = 0.8$$
$$K_n^{\frac{ATP}{ADP}} = 24$$
$$k = 2.3$$

The factor $\left(\frac{h_{mito}}{h_{in}} \right)^6$ was included to ensure proper activation of complex IV with increased demand, but complex IV activity might actually be regulated by the intra- and/or exrtramitochondrial ATP/ADP ratio.

CAC

Pyruvate exchanger [15]

(Pyr_in + H_mito <-> Pyr_mito + H_in)

$$v_{pyr-exchanger} = V_{\max}^{Pyr-exchanger} \cdot \frac{\left(Pyr_{in} \cdot H_{in} - Pyr_{mito} \cdot H_{mito}\right)}{\left(1+\frac{Pyr_{in}}{K_m^{Pyr_{in}}}\right)\left(1+\frac{Pyr_{mito}}{K_m^{Pyr_{mito}}}\right)}$$

$$K_m^{Pyr_{in}} = 0.15$$
$$K_m^{Pyr_{mito}} = 0.15$$

Pyruvatedehydrogenase complex [16], [17]

(Pyr_mito + CoA + NAD -> AcCoA + Co2 + NADH)

$$v_{pdhc} = V_{\max}^{pdhc}\left(1 + A_{\max}^{Ca_{mito}} \frac{Ca_{mito}}{Ca_{mito} + K_a^{Ca_{mito}}}\right)\left(\frac{Pyr_{mito}}{Pyr_{mtio} + K_m^{Pyr}}\right)\left(\frac{NAD_{mito}}{NAD_{mito} + K_m^{NAD_{mito}}}\right)\left(\frac{CoA_{mito}}{CoA_{mito} + K_m^{CoA_{mito}}}\right)$$

$$A_{\max}^{Ca_{mito}} = 1.7$$
$$K_a^{Ca_{mito}} = 10^{-3}$$
$$K_m^{Pyr} = 0.090$$
$$K_m^{NAD_{mito}} = 0.036$$
$$K_m^{CoA_{mito}} = 0.0047$$

Citrate synthase [18],

(Oxa + AcCoA -> Cit)

$$v_{cs} = V_{\max}^{cs}\left(\frac{Oxa_{mtio}}{Oxa_{mito} + K_m^{Oxa}\left(1+\frac{Cit_{mito}}{3.7}\right)}\right)\left(\frac{AcCoA_{mito}}{AcCoA_{mito} + K_m^{AcCoA} \cdot \left(1+\frac{CoA_{mito}}{0.025}\right)}\right)$$

$$K_m^{Oxa} = 0.0045$$
$$K_m^{AcCoA} = 0.005$$

$$K_i^{Cit} = 3.7$$
$$K_i^{CoA} = 0.025$$

Aconitase [19],

(Cit <-> IsoCit)

$$v_{Aco} = V_{\max}^{Aco} \frac{Cit_{mito} - \frac{IsoCit_{mito}}{K_{eq}^{Aco}}}{1 + \frac{Cit_{mito}}{K_m^{Cit}} + \frac{IsoCit_{mito}}{K_m^{IsoCit}}}$$

$$K_{eq}^{Aco} = 0.067$$
$$K_m^{Cit} = 0.48$$
$$K_m^{IsoCit} = 0.12$$

NAD-dependent isocitrate dehydrogenase [20] [21] [22] [23] [24]

(IsoCit + NAD -> akg + NADH)

$$v_{icdh} = V_{\max}^{icdh} \left(\frac{IsoCit_{mito}^{n^{IsoCit}}}{IsoCit_{mito}^{n^{IsoCit}} + \left(K_m^{IsoCit}\right)^{n^{IsoCit}}} \right) \left(\frac{NAD_{mito}}{NAD_{mito} + K_m^{NAD} \cdot \left(1 + \frac{NADH_{mito}}{K_i^{NADH}}\right)} \right)$$

$$n^{IsoCit} = 1.9$$

$$K_m^{IsoCit} = \frac{K_{m1}^{IsoCit}}{\left(1 + \left(\frac{Ca_{mito}}{K_a^{Ca}}\right)^{n^{Ca}}\right)} + K_{m2}^{IsoCit}$$

$$K_{m1}^{IsoCit} = 0.11$$
$$K_{m2}^{IsoCit} = 0.06$$
$$K_a^{Ca} = 0.0074$$
$$n^{Ca} = 2$$

$$K_m^{NAD} = 0.091$$
$$K_i^{NADH} = 0.0041$$

α-ketogluterate dehydrogenase [25-29], [27], [28], [26],

(akg + NAD + CoA -> SucCoA + NADH)

$$v_{akdhc} = V_{\max}^{akdhc} \left(1 - \frac{Ca_{mito}}{Ca_{mito} + K_i^{Ca}}\right) \left(\frac{akg_{mito}}{akg_{mito} + K_m^{akdhc}}\right) \left(\frac{NAD_{mito}}{NAD_{mito} + K_m^{NAD} \cdot \left(1 + \frac{NADH_{mito}}{K_i^{NADH}}\right)} \right) \left(\frac{CoA}{CoA + K_m^{CoA} \cdot \left(1 + \frac{SucCoA}{K_i^{SucCoA}}\right)} \right)$$

$$K_m^{akdhc} = \left(\frac{K_{m1}^{akdhc}}{\left(1 + \frac{Ca_{mito}}{K_{i-akg}^{Ca}}\right)} + K_{m2}^{akgh} \right)\left(1 + \frac{NADH_{mito}}{K_{i-akg}^{NADH}}\right)$$

$$K_{m1}^{akdhc} = 2.5$$
$$K_m^{NAD} = 0.021$$
$$K_i^{NADH} = 0.0045$$
$$K_m^{CoA} = 0.0013$$
$$K_i^{SucCoA} = 0.0045$$

Succinyl-CoA Synthetase [30], [31], [32],[33]

(SucCoA + ADP + P <-> Succ + CoA + ATP)

$$K_{eq-succoas} = 3.63$$

$$v_{succoas-atp} = V_{\max-succoas-atp}\left(1 + A_{\max}^{P} \cdot \left(\frac{P_{mito}^{n^P}}{P_{mito}^{n^P} + \left(K_m^P\right)^{n^P}}\right)\right)\left(\frac{SucCoA_{mito} \cdot ADP_{mito} \cdot P_{mito} - \frac{Succ_{mito} \cdot CoA_{mito} \cdot ATP_{mito}}{K_{eq-succoas}}}{\left(1 + \frac{SucCoA_{mito}}{K_m^{SucCoA}}\right)\left(1 + \frac{ADP_{mito}}{K_m^{ADP}}\right)\left(1 + \frac{P_{mito}}{K_m^P}\right) + \left(1 + \frac{Succ_{mito}}{K_m^{Succ}}\right)\left(1 + \frac{CoA_{mito}}{K_m^{CoA}}\right)\left(1 + \frac{ATP_{mito}}{K_m^{ATP}}\right) - 1}\right)$$

$$A_{\max}^{P} = 1.2$$
$$K_m^P = 2.5$$
$$n^P = 3$$
$$K_m^{SucCoA} = 0.041$$
$$K_m^{ADP} = 0.25$$
$$K_m^P = 0.72$$
$$K_m^{Succ} = 1.6$$
$$K_m^{CoA} = 0.056$$
$$K_m^{ATP} = 0.017$$

Succinatedehydrogenase [34], [35]

(Succ + Q <-> Fum + QH$_2$)

$$v_{succdh} = V_{\max-succdh}\left(\frac{Succ_{mito} \cdot Q_n - \frac{Fum_{mito} \cdot (QH_2)_n}{K_{eq-succdh}}}{Succ_{mito} + K_m^{Succ}\left(1 + \frac{Mal_{mito}}{K_i^{Mal}}\right)}\right)$$

$$K_{eq-succdh} = \exp\left(\frac{25 \cdot F}{R \cdot T}\right)$$

$$K_m^{Succ} = 1.6$$

$$K_i^{Mal} = 2.2$$

Fumerase [36, 37]

(Fum <-> Mal)

$$v_{fum} = V_{\max-fum}\left(\frac{Fum_{mito} - \frac{Mal_{mito}}{K_{eq-fum}}}{1 + \frac{Fum_{mito}}{K_m^{Fum}} + \frac{Mal_{mito}}{K_m^{Mal}}}\right)$$

$$K_{eq-fum} = 4.4$$
$$K_m^{Fum} = 0.14$$

$$K_m^{Mal} = 0.3$$

Malatedehydrogenase [24], [38], [39]

(Mal + NAD < -> Oxa + NADH)

$$v_{mdh} = V_{\max-mdh}\left(\frac{Mal_{mito}\cdot NAD_{mito} - \frac{Oxa_{mito}\cdot NADH_{mito}}{K_{eq-mdh}}}{\left(1+\frac{Mal_{mito}}{K_m^{Mal}}\right)\left(1+\frac{NAD_{mito}}{K_m^{NAD}}\right)+\left(1+\frac{Oxa_{mito}}{K_m^{Oxa}}\right)\left(1+\frac{NADH_{mito}}{K_m^{NADH}}\right)-1}\right)$$

$$K_{eq-mdh} = 1.2\cdot 10^{-3}$$
$$K_m^{Mal} = 0.145$$
$$K_m^{NAD} = 0.06$$
$$K_m^{Oxa} = 0.017$$
$$K_m^{NADH} = 0.044$$

1. **Berg, J. M., J. L. Tymoczko, and L. Stryer. 2007. Biochemistry. Freeman, New York, N.Y.**
2. **Junge, W., B. Rumberg, and H. Schroder. 1970. The necessity of an electric potential difference and its use for photophosphorylation in short flash groups. Eur J Biochem 14:575-581.**

3. Bohnensack, R. 1982. The role of the adenine nucleotide translocator in oxidative phosphorylation. A theoretical investigation on the basis of a comprehensive rate law of the translocator. J Bioenerg Biomembr 14:45-61.
4. Nicolli, A., A. Redetti, and P. Bernardi. 1991. The K+ conductance of the inner mitochondrial membrane. A study of the inducible uniport for monovalent cations. J Biol Chem 266:9465-9470.
5. Rolfe, D. F. S., A. J. Hulbert, and M. D. Brand. 1994. Characteristics of Mitochondrial Proton Leak and Control of Oxidative-Phosphorylation in the Major Oxygen-Consuming Tissues of the Rat. Biochimica Et Biophysica Acta-Bioenergetics 1188:405-416.
6. Krishnamoorthy, G., and P. C. Hinkle. 1984. Non-Ohmic Proton Conductance of Mitochondria and Liposomes. Biochemistry 23:1640-1645.
7. Bernardi, P. 1999. Mitochondrial transport of cations: Channels, exchangers, and permeability transition. Physiological Reviews 79:1127-1155.
8. Zhang, H., S. E. Chobot, A. Osyczka, C. A. Wraight, P. L. Dutton, and C. C. Moser. 2008. Quinone and non-quinone redox couples in Complex III. J Bioenerg Biomembr 40:493-499.
9. Hendler, R. W., B. Bunow, and J. S. Rieske. 1985. Thermodynamic and kinetic considerations of Q-cycle mechanisms and the oxidant-induced reduction of cytochromes b. J Bioenerg Biomembr 17:51-64.
10. Demin, O. V., Gorianin, II, B. N. Kholodenko, and H. V. Westerhoff. 2001. [Kinetic modeling of energy metabolism and generation of active forms of oxygen in hepatocyte mitochondria]. Mol Biol (Mosk) 35:1095-1104.
11. Brown, G. C., and M. D. Brand. 1988. Proton/electron stoichiometry of mitochondrial complex I estimated from the equilibrium thermodynamic force ratio. Biochem J 252:473-479.
12. Long, J., F. Gao, L. Tong, C. W. Cotman, B. N. Ames, and J. Liu. 2009. Mitochondrial decay in the brains of old rats: ameliorating effect of alpha-lipoic acid and acetyl-L-carnitine. Neurochem Res 34:755-763.
13. Arnold, S., and B. Kadenbach. 1999. The intramitochondrial ATP/ADP-ratio controls cytochrome c oxidase activity allosterically. FEBS Lett 443:105-108.
14. Napiwotzki, J., and B. Kadenbach. 1998. Extramitochondrial ATP/ADP-ratios regulate cytochrome c oxidase activity via binding to the cytosolic domain of subunit IV. Biol Chem 379:335-339.
15. Halestrap, A. P. 1975. The mitochondrial pyruvate carrier. Kinetics and specificity for substrates and inhibitors. Biochem J 148:85-96.
16. Blass, J. P., and C. A. Lewis. 1973. Kinetic properties of the partially purified pyruvate dehydrogenase complex of ox brain. Biochem J 131:31-37.
17. Land, J. M., and J. B. Clark. 1973. Effect of phenylpyruvate on pyruvate dehydrogenase activity in rat brain mitochondria. Biochem J 134:539-544.
18. Matsuoka, Y., and P. A. Srere. 1973. Kinetic studies of citrate synthase from rat kidney and rat brain. J Biol Chem 248:8022-8030.
19. Guarriero-Bobyleva, V., M. A. Volpi-Becchi, and A. Masini. 1973. Parallel partial purification of cytoplasmic and mitochondrial aconitate hydratases from rat liver. Eur J Biochem 34:455-458.
20. Rutter, G. A., and R. M. Denton. 1988. Regulation of NAD+-linked isocitrate dehydrogenase and 2-oxoglutarate dehydrogenase by Ca2+ ions within toluene-permeabilized rat heart mitochondria. Interactions with regulation by adenine nucleotides and NADH/NAD+ ratios. Biochem J 252:181-189.

21. Willson, V. J., and K. F. Tipton. 1980. Allosteric properties of ox brain nicotinamide--adenine dinucleotide dependent isocitrate dehydrogenase. J Neurochem 34:793-799.

22. Rutter, G. A., and R. M. Denton. 1989. Rapid purification of pig heart NAD+-isocitrate dehydrogenase. Studies on the regulation of activity by Ca2+, adenine nucleotides, Mg2+ and other metal ions. Biochem J 263:445-452.

23. Willson, V. J., and K. F. Tipton. 1979. Purification and characterization of ox brain NAD+-dependent isocitrate dehydrogenase. J Neurochem 33:1239-1247.

24. Lai, J. C., and J. B. Clark. 1978. Isocitrate dehydrogenase and malate dehydrogenase in synaptic and non-synaptic rat brain mitochondria: a comparison of their kinetic constants. Biochem Soc Trans 6:993-995.

25. McCormack, J. G., and R. M. Denton. 1979. The effects of calcium ions and adenine nucleotides on the activity of pig heart 2-oxoglutarate dehydrogenase complex. Biochem J 180:533-544.

26. Smith, C. M., J. Bryla, and J. R. Williamson. 1974. Regulation of mitochondrial alpha-ketoglutarate metabolism by product inhibition at alpha-ketoglutarate dehydrogenase. J Biol Chem 249:1497-1505.

27. Lai, J. C., and A. J. Cooper. 1986. Brain alpha-ketoglutarate dehydrogenase complex: kinetic properties, regional distribution, and effects of inhibitors. J Neurochem 47:1376-1386.

28. Luder, A. S., J. K. Parks, F. Frerman, and W. D. Parker, Jr. 1990. Inactivation of beef brain alpha-ketoglutarate dehydrogenase complex by valproic acid and valproic acid metabolites. Possible mechanism of anticonvulsant and toxic actions. J Clin Invest 86:1574-1581.

29. Faff-Michalak, L., and J. Albrecht. 1993. The two catalytic components of the 2-oxoglutarate dehydrogenase complex in rat cerebral synaptic and nonsynaptic mitochondria: comparison of the response to in vitro treatment with ammonia, hyperammonemia, and hepatic encephalopathy. Neurochem Res 18:119-123.

30. Phillips, D., A. M. Aponte, S. A. French, D. J. Chess, and R. S. Balaban. 2009. Succinyl-CoA synthetase is a phosphate target for the activation of mitochondrial metabolism. Biochemistry 48:7140-7149.

31. Johnson, J. D., W. W. Muhonen, and D. O. Lambeth. 1998. Characterization of the ATP- and GTP-specific succinyl-CoA synthetases in pigeon. The enzymes incorporate the same alpha-subunit. J Biol Chem 273:27573-27579.

32. Krivanek, J., and L. Novakova. 1991. A novel effect of vanadium ions: inhibition of succinyl-CoA synthetase. Gen Physiol Biophys 10:71-82.

33. Lynn, R., and R. W. Guynn. 1978. Equilibrium constants under physiological conditions for the reactions of succinyl coenzyme A synthetase and the hydrolysis of succinyl coenzyme A to coenzyme A and succinate. J Biol Chem 253:2546-2553.

34. Vinogradov, A. D., A. B. Kotlyar, V. I. Burov, and Y. O. Belikova. 1989. Regulation of succinate dehydrogenase and tautomerization of oxaloacetate. Adv Enzyme Regul 28:271-280.

35. Gutman, M., and N. Silman. 1975. The steady state activity of succinate dehydrogenase in the presence of opposing effectors.II. Reductive activation of succinate dehydrogenase in presence of oxaloacetate. Mol Cell Biochem 7:177-185.

36. Kobayashi, K., T. Yamanishi, and S. Tuboi. 1981. Physicochemical, catalytic, and immunochemical properties of fumarases crystallized separately from mitochondrial and cytosolic fractions of rat liver. J Biochem 89:1923-1931.

37. Bock, R. M., and R. A. Alberty. 1953. Studies of the Enzyme Fumarase .1. Kinetics and Equilibrium. Journal of the American Chemical Society 75:1921-1925.

38. Gelpi, J. L., A. Dordal, J. Montserrat, A. Mazo, and A. Cortes. 1992. Kinetic studies of the regulation of mitochondrial malate dehydrogenase by citrate. Biochem J 283 (Pt 1):289-297.

39. Jespersen, N. 1976. Thermochemistry of Reaction Catalyzed by Malate Dehydrogenase. Thermochimica Acta 17:23-27.
